# RRM1 and ERCC1 as biomarkers in patients with locally advanced and metastatic malignant pleural mesothelioma treated with continuous infusion of low-dose gemcitabine plus cisplatin

**DOI:** 10.1186/s12885-021-08287-5

**Published:** 2021-08-05

**Authors:** Wendy Muñoz-Montaño, Sae Muñiz-Hernández, Alejandro Avilés-Salas, Rodrigo Catalán, Luis Lara-Mejía, Suraj Samtani-Bassarmal, Andres F. Cardona, Jorge Mendoza-Desión, Daniel Hernández-Cueto, Altagracia Maldonado, Guillermina Baay-Guzmán, Sara Huerta-Yepes, Oscar Arrieta

**Affiliations:** 1 Thoracic Oncology Unit, Instituto Nacional de Cancerología, San Fernando 22 Sección XVI, Tlalpan, 14080 Mexico City, Mexico; 2 Laboratory of Personalized Medicine, Instituto Nacional de Cancerología, San Fernando 22 Sección XVI, Tlalpan, 14080 Mexico City, Mexico; 3 Department of Pathology, Instituto Nacional de Cancerología, Mexico City, Mexico; 4 Department of Oncology, Bradford Hill, Santiago, Chile; 5 Foundation for Clinical and Applied Cancer Research (FICMAC), Bogotá, Colombia; 6 Clinical and Translational Oncology Group, Clínica del Country, Bogotá, Colombia; 7 Molecular Markers Laboratory, Hospital Infantil de México Federico Gómez, Secretaría de Salud, Mexico City, Mexico

**Keywords:** RRM1, ERCC1, Immunohistochemistry, Biomarkers, Mesothelioma

## Abstract

**Background:**

Malignant Pleural Mesothelioma (MPM) is a rare but aggressive neoplasia that usually presents at advanced stages. Even though some advances have been achieved in the management of patients with MPM, this malignancy continuous to impose a deleterious prognosis for affected patients (12–18 months as median survival, and 5–10% 5-year survival rate), accordingly, the recognition of biomarkers that allow us to select the most appropriate therapy are necessary.

**Methods:**

Immunohistochemistry semi-quantitative analysis was performed to evaluate four different biomarkers (ERCC1, RRM1, RRM2, and hENT-1) with the intent to explore if any of them was useful to predict response to treatment with continuous infusion gemcitabine plus cisplatin. Tissue biopsies from patients with locally advanced or metastatic MPM were analyzed to quantitatively asses the aforementioned biomarkers. Every included patient received treatment with low-dose gemcitabine (250 mg/m^2^) in a 6-h continuous infusion plus cisplatin 35 mg/m^2^ on days 1 and 8 every 3 weeks as first-line therapy.

**Results:**

From the 70 eligible patients, the mean and standard deviation (SD) for ERCC1, RRM1, RRM2 and hENT-1 were 286,178.3 (**±** 219, 019.8); 104,647.1 (**±** 65, 773.4); 4536.5 (**±** 5, 521.3); and 2458.7 (**±** 4, 983.4), respectively. Patients with high expression of RRM1 had an increased median PFS compared with those with lower expression (9.5 vs 4.8 months, *p* = < 0.001). Furthermore, high expression of RRM1 and ERCC1 were associated with an increased median OS compared with their lower expression counterparts; [(23.1 vs 7.2 months for RRM1 *p* = < 0.001) and (17.4 vs 9.8 months for ERCC1 *p* = 0.018)].

**Conclusions:**

ERCC1 and RRM1 are useful biomarkers that predict better survival outcomes in patients with advanced MPM treated with continuous infusion of gemcitabine plus cisplatin.

## Background

Malignant pleural mesothelioma (MPM) is rare neoplasia highly correlated with asbestos and erionite exposure [1]. Even though occupational exposure to asbestos is progressively decreasing, the incidence and mortality due to MPM are still increasing [1]. According to the World Health Organization (WHO), MPM was responsible for 38,000 deaths worldwide between 1994 and 2008. Interestingly, higher incidence and mortality rates were reported in developed countries such as the United States of America, the United Kingdom, and Japan [2]. In Mexico, MPM is the 34th most frequently diagnosed neoplasia; however, since it is commonly diagnosed in advanced stages, some studies suggest that up to 70% of cases might remain undiagnosed or unreported [3]. During the last decade, different treatments for MPM have shown some advances; unfortunately, this has not been translated into significantly better outcomes. The combination of pemetrexed plus a platinum agent currently stands as the first-line therapy approved by the US Food and Drug Administration (FDA) to treat advanced MPM. Treatment with this combination is reported to yield 6 months median progression-free survival (PFS), and approximately 15.3 months median overall survival (OS) [4]. In 2014, our group reported a remarkable clinical efficacy using combination chemotherapy with low-dose gemcitabine administered in a six-hour continuous infusion plus cisplatin as first-line therapy for patients with advanced MPM [5].

Great efforts had been made to identify biomarkers that allow us to predict treatment response in patients with MPM; however, currently there is no widespread accepted biomarker or model for predicting therapeutic response in patients with advanced MPM [6–8].

Ribonucleotide reductase is an enzyme responsible for de novo synthesis of most deoxyribonucleotides, the abundance of two subunits of this enzyme, named “Ribonucleotide Reductase M1 & M2” (RRM1 & M2), correlate with the ability to repair DNA in cancerous cells [9]. In a similar fashion, Excision repair cross complementation group 1 (ERCC1) performs an essential role in several DNA repair pathways [10]. These molecules (RRM1-M2 and ERCC1) have been proposed as biomarkers to predict treatment response to chemotherapy schemes in patients with several solid tumors [10–17]. However, there is insufficient evidence to postulate either of these molecules as an undisputable biomarker for treatment response in patients with MPM. Another molecule named “human equilibrative nucleoside transporter-1” (hENT-1), which is a nucleoside transporter protein that mediates cellular entry of cytotoxic agents used as chemotherapics, had also rose interest as a potential biomarker of response to chemotherapeutic regimens, including those containing gemcitabine [12, 18, 19]. Albeit, its role as a biomarker in MPM has not been studied.

Considering the deleterious prognosis that MPM confers to affected patients and the increasing incidence of this malignancy, identifying biomarkers that aid us in predicting treatment response is a promising area for research. Therefore, our study aimed to analyze if ERCC1, RRM1, RRM2, and hENT-1 correlated with response to low-dose gemcitabine in six-hour continuous infusion plus cisplatin in patients with advanced MPM.

## Methods

### Study design

Patients with a confirmed diagnosis of advanced MPM that were treated at the Thoracic Oncology Unit of *Instituto Nacional de Cancerología* (INCan) were enrolled from January 2012 until December 2017 if they fulfilled inclusion criteria. The entire study was performed in accordance with the Declaration of Helsinki, and with the principles of good clinical practice. All patients provided written informed consent to participate, and the study was approved by the scientific and bioethical committees of INCan (010/056/ICI) (CEI/656/10).

Locally advanced or metastatic (clinical stage III-IV, according to the AJCC Cancer Staging Manual, 7th edition) newly diagnosed patients who had never received chemotherapy were eligible. Available tumor sample tissue was necessary to perform biomarkers analyses, patients without enough available tissue were excluded. Patients with cardiovascular disease (defined as: coronary artery disease, congestive heart failure or arrhythmia), abnormal renal function (serum creatinine ≥1.5 mg/dL or creatinine clearance < 60 ml/min/m^2^), abnormal hepatic function (serum bilirubin ≥1.6 mg/dL) or abnormal bone marrow function (leukocyte ≤4, 000/μL or platelet count ≤100,000/μL) were excluded.

### Samples and data collection

Tumor samples were obtained from 70 patients with a confirmed diagnosis of MPM. All tissue samples were processed to determine the expression of the RRM1, RRM2, ERCC1, and hENT-1. The clinical and demographic data were extracted from from medical records.

### Immunohistochemistry

The level of expression of ERCC1, RRM1, RRM2, and hENT-1 was determined by immunohistochemistry using the following commercial antibodies: ERCC1 (GTX22356), RRM1 (GTX100758), RRM2 (GTX103193) from Genetex Inc., USA; and hENT (SC-48489) from Santa Cruz Biotechnology, USA. Tissue biopsies were processed to obtain slices of 4 μm; next, slices were deparaffinized at 60 °C in dry heat and rehydrated in a series of xylene-ethanol solutions. Then, slices were washed with citrate buffer to increase antigen exposure. Afterward, samples were covered with primary antibody for 2 hours and then washed and incubated with a biotinylated secondary antibody (Universal LSAB kit; Dako Corporation, Carpinteria, CA, USA) for 30 min at room temperature. Next, streptavidin-horseradish peroxidase conjugate (Universal LSAB kit, Dako Corp) and 3, 3′-diaminobenzidine tetra-hydrochloride (liquid DAB, Dako Corp) were added for 30 min; reaction with diaminobenzidine was stopped by adding distilled water, and the slides were counterstained with Hematoxylin (CatHE-M, Biocare Medical, CA. USA). Finally, slices were mounted in Entellan medium (Merk Co, USA).

### Scoring of immunohistochemical staining

A semi-quantitative assessment of staining was conducted by a senior pathologist, who was blinded to clinical outcomes. Positive expression, indicated by a brown color, was quantified as follows: the total available sample was quantified in each case, only tumor cell regions were considered (if the sample contained inflammatory regions, these regions were excluded from analysis). Available sample was divided virtually into 300 μm^2^ sections, to eliminate variations, the summatory of all parts was carried out and the total number of pixels obtained was reported for each sample and each biomarker. The staining density was analyzed using the Image Pro-plus 7.0 software (MediaCybernetics, Rockville, MD, USA), which was obtained with the diffusion of light wavelength through the color density in the cells that stained brown. Both negative and positive (weakly positive, moderately positive, and strongly positive) intensities were assessed in tumoral cells. For staining digital analysis, we took into account cytoplasm and nuclear expression (RRM1, RRM2 and hENT-1 were almost exclusively localized on cytoplasm; while ERCC1 was localized at nucleus exclusively).

### Treatment

All patients received chemotherapy with low dose gemcitabine 250 mg/m^2^ administered in a six-hours continuous infusion plus cisplatin 35 mg/m^2^ on days 1 and 8 every 3 weeks for up to 6 cycles, or until disease progression, development of unacceptable toxicity, or withdrawal of informant consent. Those patients with unacceptable toxicity to cisplatin received carboplatin (AUC 5 every 3 weeks on day 1 of each cycle) without changing gemcitabine dosage.

#### Survival assessment

PFS, OS, and ORR were correlated according to the expression of the four biomarkers. PFS was defined as the period from starting treatment to disease progression or death. OS was defined as the time from diagnosis to death or loss of follow-up. The ORR was the sum of complete response and partial response assessed by a CT-scan or PET/CT every three cycles according to the modified Response Evaluation Criteria in Solid Tumors (RECIST) for MPM version 1.0 [20]. Adverse events were graded with the Common Terminology Criteria for Adverse Events, version 4.0.

#### Statistical analysis

For descriptive purposes, continuous variables were summarized as arithmetic means and SD; categorical variables were comprised as frequencies and proportions. Patients were categorized into high or low expression group based on the four biomarkers median expression. For survival analysis, all variables were dichotomized. The OS and PFS were analyzed by the Kaplan-Meier method, and comparisons among subgroups were assessed by the log-rank test. Patients who lost follow-up were censored from survival analysis. Finally, we performed a multivariate analysis with a Cox proportional model to estimate the hazard ratios with 95% CI for disease progression and death. Statistical significance was predetermined to be present for values of *p* < 0.05 based on a two-sided test. All statistical analyses were carried out using the SPSS (Statistical Package for the Social Sciences) version 24.0 (SPSS Inc., Chicago, IL).

## Results

### Study sample

From a total of 70 included patients, the median age at diagnosis was 60 years, males represented in 64.2% of population, prior exposure to asbestos was reported in 51.4% of patients, smoking history was reported in 54.3%. Most patients were diagnosed with stage IV MPM (70%); 71.4% had an ECOG-PS grade of 0–1. Epithelioid MPM was the most common histological subtype, identified in 82.9% of patients (Table 1).

**Table 1:** General characteristics of patients

	Total % (n) *N* = 70
Sex	
Male	64.2 (46)
Female	35.8 (24)
Age, years	
Mean (± SD)	60 (± 11.1)
< 60 years	47.1 (33)
≥ 60 years	52.9 (37)
Smoking history	
Present	54.3 (38)
Absent	45.7 (32)
Asbestos exposure	
Present	51.4 (36)
Absent	48.6 (34)
Wood-smoke exposure	
Absent	64.3 (45)
Present	35.7 (25)
ECOG-PS	
0–1	71.4 (50)
2	28.6 (20)
Histology	
Epithelial	82.9 (58)
Other	17.1 (12)
Karnofsky	
< 80	41.4 (29)
≥ 80	58.6 (41)
Stage disease	
III	30.0 (21)
IV	70.0 (49)

*ECOG-PS* Eastern Cooperative Oncology Group – Performance Status, *SD* Standard Deviation

### Expression level of biomarkers

**Fig. 1:**
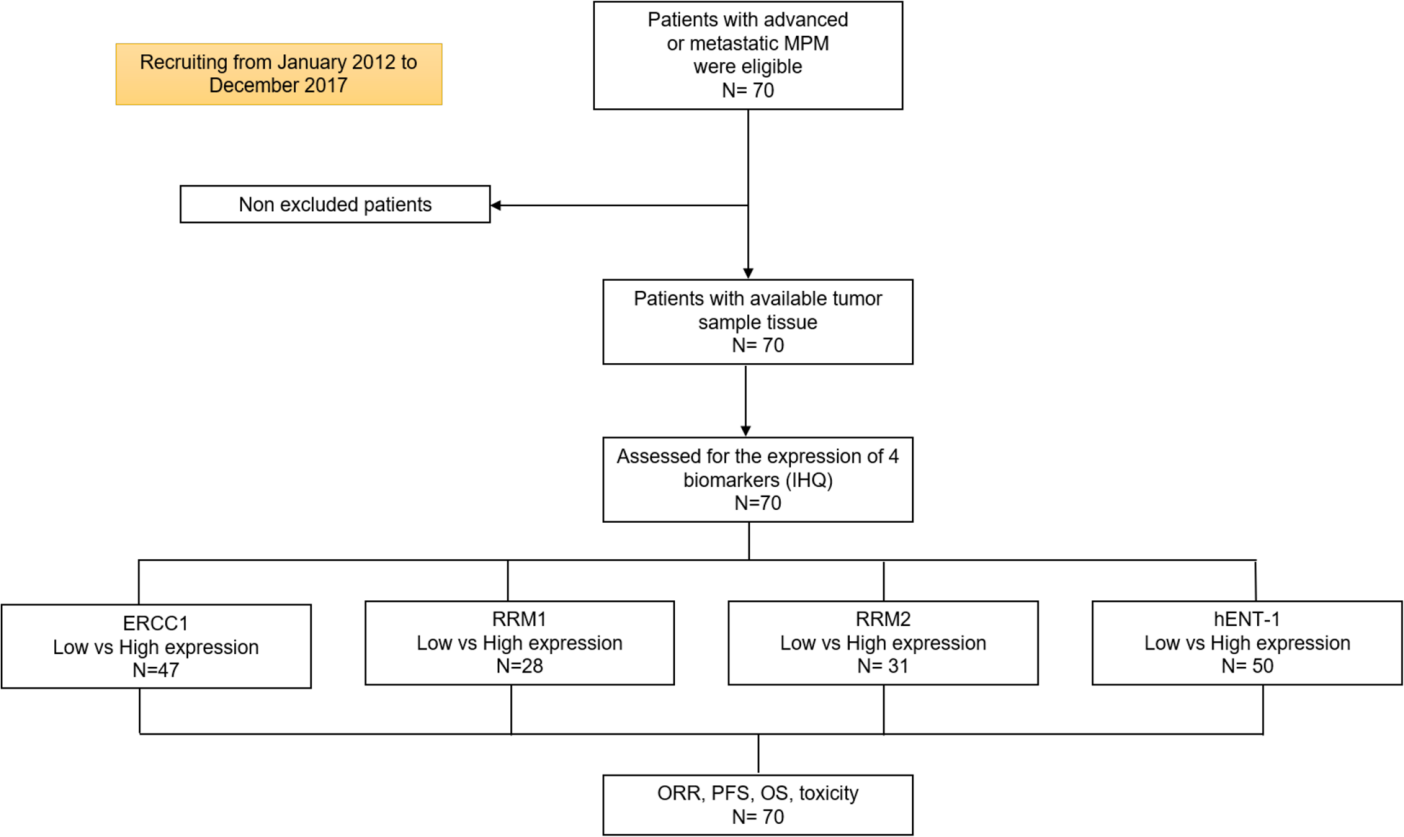
Strobe flow chart

**Fig. 2:**
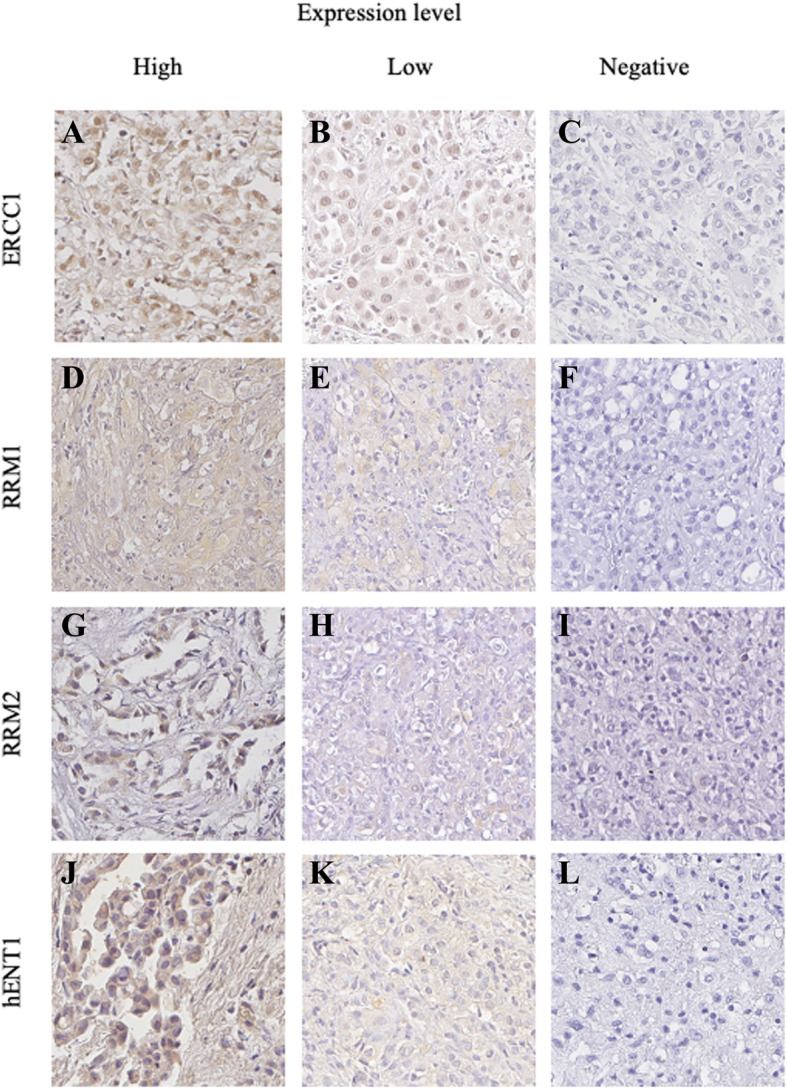
Immunohistochemical analysis for ERCC1, RRM1, RRM2, and hENT-1 expression in malignant pleural mesothelioma patients. High and low nuclear expression for ERCC1 (**a**, **b**), and high and low cytoplasmic expression for RRM1 (**d**, **e**), RRM2 (**g**, **h**), and hENT-1 (**j**, **k**), respectively. Negative controls (without primary antibody) are shown for each molecule in **c**, **f**, **i**, and **l**, respectively

We obtained tumor sample for biomarker assessment from every patient; however, enough tissue to perform IHC to analyze all biomarkers was not available in every patient (Fig. 1). Patients were classified into high or low expression groups according to the median value of expression in each biomarker. Figure 2 shows a representative image of each biomarker according to expression level (low or high). The mean and SD for expression of ERCC1, hENT-1, RRM1 and RRM2 was: 286,178.3 (± 219, 019.8); 194,647.1 (±104, 647.1); 4536.5 (± 5, 521.3) and 2458.7 (± 4, 983.4), respectively (Table 2). Distribution was similar between high and low expression groups in all four biomarkers.

**Table 2:** Total expression levels of Biomarkers (*N* = 70)

ERCC1	
Mean (± SD)	286, 178.3 (**±** 219, 019.8)
Low expression % (n)	48.9 (23)
High expression % (n)	51.1 (24)
hENT-1	
Mean (± SD)	104, 647.1 (**±** 65, 773.4)
Low expression % (n)	50 (25)
High expression % (n)	50(25)
RRM1	
Mean (± SD)	4, 536.5 (**±** 5, 521.3)
Low expression % (n)	46.4 (13)
High expression % (n)	53.6 (15)
RRM2	
Mean (± SD)	2458.7 (**±** 4, 983.4)
Low expression % (n)	48.4 (15)
High expression % (n)	51.6 (16)

Note: the total of patients varies across the different biomarkers due to the lack of tissue

*SD* Standard deviation, *ERCC1* Excision repair cross complementation-1, *hENT-1* Human equilibrative nucleoside transporter-1, *RRM1* Ribonucleotide Reductase M1, *RRM2* Ribonucleotide Reductase M2

### ORR, PFS, and OS

All patients received first-line therapy with low-dose gemcitabine (250 mg/m^2^ in a 6-h continuous infusion in days 1 and 8), plus cisplatin (35 mg/m2 in day one every 21 days) for a median number of 6 cycles [1–8, 11, 12, 20]. The overall response rate was 47.1% (33/70), and the disease control rate was 80% (56/70). No complete responses were observed (Table 3).

**Table 3:** Characteristics of cisplatin/gemcitabine treatment in the study population

General data	*N* = 70
Number of cycles	
Median (min-max)	6 (1–11)
Overall Response Rate	% (n/N)
Present	47.1 (33/70)
Absent	45.7 (32/70)
Unable to assess	7.1 (5/70)
Disease Control Rate	
Yes	80 (56)
No	12.9 (9)
Unable to assess	7.1 (5)

**Fig. 3:**
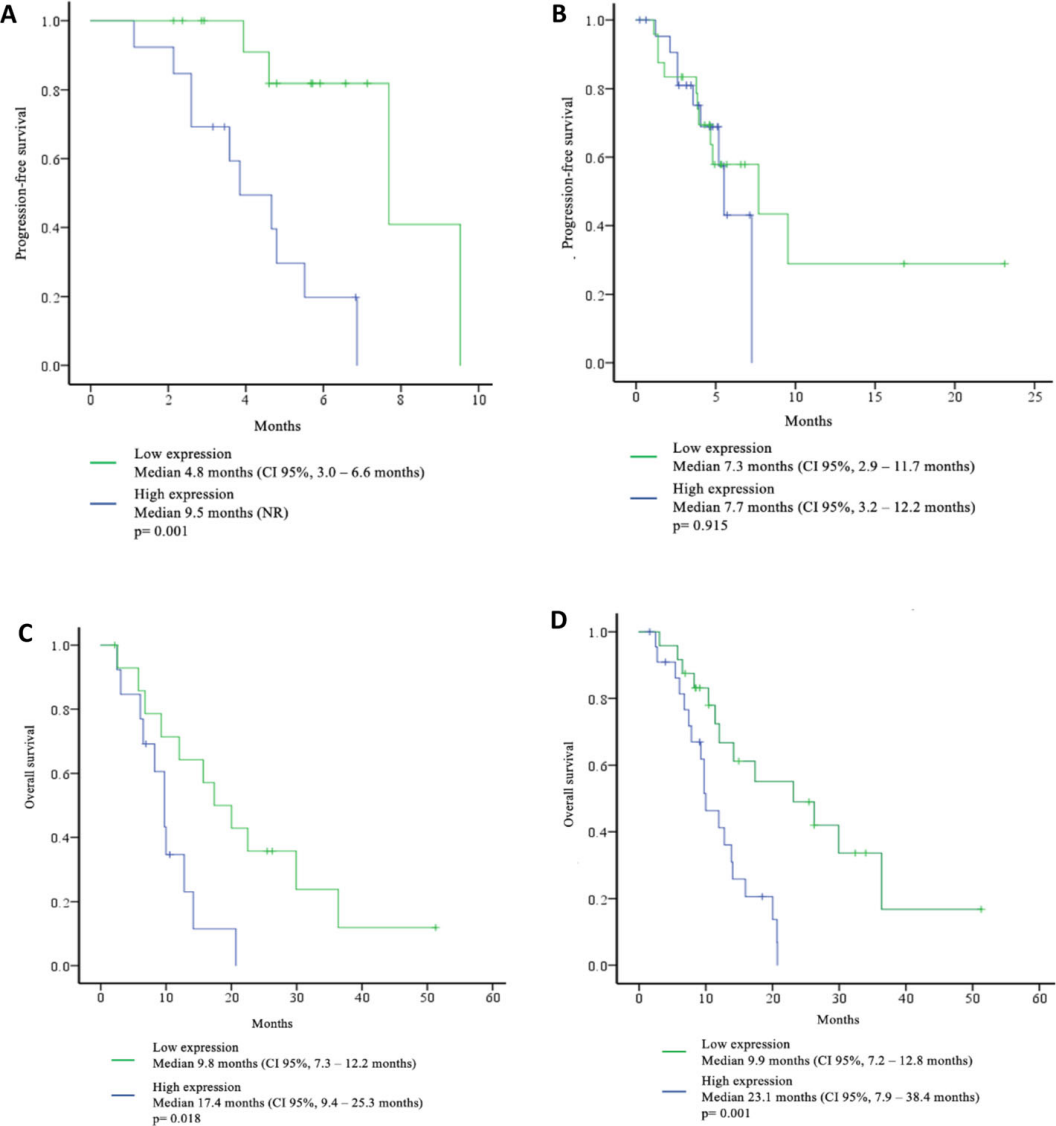
Progression-free survival (PFS) according to RRM1 (**a**) and ERCC1 (**b**) expression, high vs low. Overall survival (OS) according to RRM1 (**c**) and ERCC1 (**d**) expression, high vs low

The median PFS was 7.6 months (4.2–11.1 months). According to the univariate survival analysis, only epithelioid histology subtype was associated with an increased PFS [9.5 months 95%CI (0.0–19.9 months), *p* = 0.013]. Biomarker analysis revealed that high expression of RRM1 was correlated with an increased PFS compared with the low expression group [9.5 months vs. 4.8 months; 95%CI (NR), *p* = 0.001] (Fig. 3a). On the other hand, ERCC1 expression was not significantly associated with PFS (7.7 months vs. 7.3 months, *p* = 0.915) (Fig. 3b). At the univariate analysis, no biomarker was associated with an increased in PFS. At the multivariate analysis, high expression of RRM1 remained significantly associated with a decreased risk of progression [HR 0.11, 95% CI (0.02–0.52), *P* = 0.005] (Table 4).

**Table 4:** Unadjusted and adjusted hazard ratios for the factors associated with PFS to cisplatin/gemcitabine among patients with MPM

	HR (95% CI)	*p*-Value	HR (95% CI)	*p*-value
Sex	1.83 (0.85–3.97)	0.124	1.36 (0.46–4.01)	0.573
Age, 60+ years	1.27 (0.58–2.76)	0.552		
Tobacco exposure	1.08 (0.49–2.37)	0.843		
Exposure to asbestos	0.61 (0.28–1.33)	0.218		
ECOG PS	2.01 (0.92–4.39)	0.080		
Histology	2.90 (1.20–7.02)	**0.018**		
Karnofsky	0.61 (0.28–1.33)	0.215		
Disease stage	0.74 (0.34–1.63)	0.457		
ERCC1	0.95 (0.39–2.31)	0.915		
RRM1	0.11 (0.02–0.52)	**0.005**	0.11 (0.02–0.52)	**0.005**
RRM2	0.71 (0.24–2.09)	0.532		
hENT-1	1.13 (0.47–2.74)	0.780		

*ECOG-PS* Eastern Cooperative Oncology Group – Performance Status, *ERCC1* Excision repair cross complementation-1, *hENT-1* Human equilibrative nucleoside transporter-1, *RRM1* Ribonucleotide Reductase M1, *RRM2* Ribonucleotide Reductase M2

### OS and clinical features

The median OS was 14 months (95% CI 10.5–17.5). At the univariate analysis, high expression of RRM1 and ERCC1 were associated with an increased OS compared with those patients with a low expression; [(17.4 vs 9.8 months; *p* = 0.018) for RRM1, and (23.1 vs 7.2 months, *p* = 0.001) for ERCC1] (Fig. 3 C & D). At the multivariate analysis, a Karnofsky Performance Scale Index > 80, and high expression of ERCC1, were the only variables significantly associated with a decreased risk of death [HR 0.16, 95% CI (0.04–0.75), *p* = 0.019 and HR 0.24, 95% CI (0.07–0.79), *p* = 0.020, respectively] (Table 5).

**Table 5:** Unadjusted and adjusted hazard ratios for the factors associated with OS to cisplatin/gemcitabine treatment among patients with MPM

	HR (95% CI)	*p*-Value	HR (95% CI)	*p*-value
Sex	1.21 (0.66–2.23)	0.537		
Age, years	1.62 (0.90–2.95)	0.108	2.77 (0.75–10.3)	0.125
Tobacco exposure	0.64 (0.35–1.16)	0.141	0.82 (0.25–2.75)	0.821
Exposure to asbestos	0.72 (0.40–1.31)	0.283		
ECOG PS	1.64 (0.79–3.43)	0.186		
Histology	1.60 (0.66–3.84)	0.297		
Karnofsky	0.59 (0.32–1.11)	0.102	0.16 (0.04–0.75)	**0.019**
Disease stage	0.99 (0.53–1.87)	0.979		
ORR	2.17 (1.25–3.78)	**0.006**	1.55 (0.71–3.36)	0.269
ERCC1	0.28 (0.12–0.63)	**0.002**	0.24 (0.07–0.79)	**0.020**
RRM1	0.32 (0.12–0.86)	**0.023**		
RRM2	0.60 (0.23–1.41)	0.246		
hENT-1	0.68 (0.34–1.39)	0.290		

*ECOG-PS* Eastern Cooperative Oncology Group – Performance Status, *ERCC1* Excision repair cross complementation-1, *hENT-1* Human equilibrative nucleoside transporter-1, *RRM1* Ribonucleotide Reductase M1, *RRM2* Ribonucleotide Reductase M2

### Toxicity

Toxicity was assessed in every patient; frequencies and severity of adverse events (AEs) were graded according to the CTCAE V4.0 and reported in Table 6. The most frequent AEs were fatigue, nausea, and vomiting, occurring in 75.7, 74.3, and 25% of the patients. The most common grade 3 AEs were neutropenia and nausea in 4 and 3%, respectively. No grade 4 or death-related events were observed.

**Table 6:** Adverse events (*N* = 70)

	Any grade % (n)	G1% (n)	G2% (n)	G3% (n)	G4% (n)
Diarrhea	21.4 (15)	17.1 (12)	4.3 (3)	–	–
Nausea	74.3 (52)	35.7 (25)	34.3 (24)	4.3 (3)	–
Vomit	35.7 (25)	24.3 (17)	8.6 (6)	2.9 (2)	–
Fatigue	75.7 (53)	40 (28)	34.4 (24)	1.4 (1)	–
Constipation	7.1 (5)	5.7 (4)	1.4 (1)	–	–
Leukopenia	20 (14)	14.3 (10)	4.3 (3)	1.4 (1)	–
Neutropenia	20 (14)	5.7 (4)	8.6 (6)	5.7 (4)	–
Neuropathy	20 (14)	11.4 (8)	7.1 (5)	1.4 (1)	–
Appetite loss	8.6 (6)	4.3 (3)	4.3 (3)	–	–

## Discussion

There are none biomarkers that have consistently demonstrated efficacy as predictors of treatment response in patients with MPM. ERCC1, RRM1, RRM2, and hENT-1 have been proposed as potential predictive biomarkers of treatment response in several types of tumors, including lung, pancreatic, ovarian, and colorectal cancer [10–16, 18]. Albeit, none of these biomarkers have been widely accepted to be useful in patients with MPM. This study aimed to correlate the expression of four biomarkers with survival outcomes in patients with unresectable MPM treated with low dose continuous infusion of gemcitabine plus cisplatin.

### RRM1 and RRM2

Ribonucleotide Reductase (RR) is a multimeric enzyme responsible for ribonucleoside diphosphate conversion to deoxyribonucleoside diphosphates. This substrate of the DNA polymerases is responsible for the synthesis of de novo deoxynucleotides. RR consists of two subunits; the large subunit M1 (RRM1), which is the regulatory one, and the small subunit M2 (RRM2), which has catalytic functions; combined, these two subunits form the active enzyme [9]. Some therapeutic agents, such as gemcitabine, are directed toward inhibiting the activity of RR. Accordingly, several trials have studied the role of RRM1 expression and the response of patients under treatment with gemcitabine.

Moreover, RRM1 has been reported as a biomarker of treatment response in several neoplasms, such as MPM, non-small cell lung cancer and pancreatic adenocarcinoma; however, results from these studies are not conclusive about the role of RRM1 as a biomarker for treatment response in patients with MPM [11, 16, 18]. Furthermore, there is evidence suggesting that the utility of RRM1 as a biomarker might depend on the therapeutic regimen employed. Zimling ZG et al. previously reported that a lower expression of RRM1 was associated with a better prognosis [21]. In contrast, our current results suggests that higher expression of RRM1 is associated with increased PFS and OS, being almost twice as high in the group with high expression compared with the group that expressed lower levels of RRM1. These results discrepancies could be explained due to the therapeutic regimen used in each study. Patients enrolled at Zimling ZG et al. study received either pemetrexed–platinum doublet or vinorelbine-platinum doublet, and only patients that presented a high expression of RRM1 and received vinorelbine showed better response (47.1% vs. 13.3%, negative vs. positive) [21]. Recently, Zito Marino et al.*,* reported thar RRM1 and ERCC1 co-expression was significantly related with worse outcomes in patients with MPM [22]. Even though results from both of these studies contrast with those reported in our present study, it should be underscored that chemotherapy schemes were different to the one we employed. This observation strengthens the hypothesis that the prognostic significance of RRM1 might vary according to the employed chemotherapy. Supporting our results, Frischknecht L et al.*,* reported an increased freedom from recurrence (FFR), and a better OS in patients with MPM and high expression of RRM-1 that received platinum-gemcitabine doublet as first line of therapy [23]. Moreover, in 2014 Szulkin et al.*,* reported that the proportion of malignant cells and RRM1 reactivity in the pleural effusions of patients with MPM correlate to drug sensitivity and survival time, therefore analyzing RRM1 in pleural effusion might be a plausible way for determining which is the best therapy in the first-line setting [24].

### ERCC1

ERCC1 has a cornerstone role in DNA repair pathways; however, similar to what happens with RRM1, the prognostic role of ERCC1 is uncertain considering previous contradictory results. Evidence has correlated negative ERCC1 expression with increased FFR compared with its positive counterpart in patients with MPM treated with platinum-based chemotherapy [23]. On the same line Zimling ZG et al.*,* reported a significant correlation between negative ERCC1 expression and increased PFS; however, differences were only seen in PFS and did not prevailed when analyzing OS [25]. Two other studies involving MPM patients treated with either pemetrexed as monotherapy, or combined with platinum did not found any association between ERCC1 expression and treatment response [26, 27]. Another trial presented by Cihan et al. studied patients treated with a combination of platinum plus pemetrexed in the first line setting, it was concluded that patients with positive ERCC1expression presented shorter OS than patients with negative ERCC1 expression (11.7 vs. 19.2 months, respectively). In the same study, a survival analysis at 1 and 2 years reported higher survival rates in patients with no expression of ERCC1 when compared with patients with positive ERCC1 expression (64 and 49% vs. 40 and 0%, respectively) [28]. Remarkably, we found opposite results, with high ERCC1 expression being marker of increased survival. In our study median OS of patients with high ERCC1 was 23.1 months, considerably higher than its counterpart (7.2 months). One of the main differences between the present study and Cihan and colleagues’ work is the therapeutic regimen utilized (platinum + pemetrexed vs platinum + gemcitabine). In line with our results, Kao et al.*,* reported that high ERCC1 expression was correlated with longer OS (27.6 vs. 10.3 months; *p* = 0.06); however, at their study patients underwent surgery (extra pleural pneumectomy) as part of the primary treatment, while in our study surgery was not considered as part of the cornerstone treatment [29].

We consider that there are two major explanations for the discrepancies observed, the first one is that chemotherapy scheme differ among studies; as previously discussed for RRM1, there is a reasonable possibility that ERCC1 perform different according to the therapy employed; another plausible explanation for the discrepancies observed among studies might be the method employed to quantify ERCC1; while we used the median staining value as the cut-off point for determining high vs low expression, most of the aforementioned studies only used positive vs negative expression for stratifying patients. Accordingly, results are inconsistent among studies, with most studies considering high ERCC1 expression as a factor of worse prognosis [22, 30, 31].

### hENT-1

Several studies have reported that hENT-1 expression could be used as a biomarker of treatment response in patients with gastrointestinal cancer who receive treatment with gemcitabine [12, 18, 19]. To our knowledge, the present study is the first to evaluate hENT1 as a biomarker for patients with MPM. Unfortunately, we found no association between hENT-1 expression levels and survival outcomes.

### Limitations

Our study had some limitations, the first one is the relatively small sample size; furthermore, we were unable to obtain enough tissue to evaluate the four proposed biomarkers in the entire population, and this should be considered a significant limitation. Unfortunately, no other study has reported a predefined wll established cut-off value to evaluate the aforementioned biomarkers, further complicating the interpretation of our results and making difficult their comparison with prior studies.

## Conclusion

In patients with advanced MPM that received first line therapy with continuous infusion of low-dose gemcitabine plus cisplatin, the higher expression of RRM1 and ERCC1 are associated with better OS. Furthermore, high expression of RRM1 is also associated with an increased PFS.

## Data Availability

Data generated from this study is available through a reasonable request to corresponding authors.

## Abbreviations

MPM: Malignant pleural mesothelioma
WHO: World Health Organization
OS: Overall survival
FDA: Food and Drug Administration
PFS: Progression free survival
RRM1 and M2: Ribonucleotide Reductase M1 & M2
ERCC1: Excision repair cross complementation group 1
hENT-1: human equilibrative nucleoside transporter-1
INCan: *Instituto Nacional de Cancerología*
ORR: Objective response rate
RECIST: Response Evaluation Criteria in Solid Tumors
EORTC: European Organization for Research and Treatment of Cancer
CALGB: Cancer and Leukemia Group B
